# Regional Variation of Extended-Spectrum Beta-Lactamase (ESBL)-Producing Enterobacterales, Fluoroquinolone-Resistant *Salmonella enterica* and Methicillin-Resistant *Staphylococcus aureus* Among Febrile Patients in Sub-Saharan Africa

**DOI:** 10.3389/fmicb.2020.567235

**Published:** 2020-09-25

**Authors:** Rehema Moraa Moirongo, Eva Lorenz, Nyanda E. Ntinginya, Denise Dekker, José Fernandes, Jana Held, Maike Lamshöft, Frieder Schaumburg, Chacha Mangu, Lwitiho Sudi, Ali Sie, Aurelia Souares, Norbert Heinrich, Andreas Wieser, Benjamin Mordmüller, Ellis Owusu-Dabo, Akim Ayola Adegnika, Boubacar Coulibaly, Jürgen May, Daniel Eibach

**Affiliations:** ^1^Infectious Disease Epidemiology, Bernhard Nocht Institute for Tropical Medicine, Hamburg, Germany; ^2^German Center for Infection Research (DZIF), Hamburg-Lübeck-Borstel-Riems, Heidelberg, Munich, Tübingen, Germany; ^3^Institute of Medical Biostatistics, Epidemiology and Informatics, University Medical Centre of the Johannes Gutenberg University Mainz, Mainz, Germany; ^4^National Institute for Medical Research-Mbeya Medical Research Center, Mbeya, Tanzania; ^5^Centre de Recherches Médicales de Lambaréné (CERMEL), Lambaréné, Gabon; ^6^Institut für Tropenmedizin, Universitätsklinikum Tübingen, Tübingen, Germany; ^7^Institute of Medical Microbiology, University Hospital Münster, Münster, Germany; ^8^Centre de Recherche en Santé de Nouna, Nouna, Burkina Faso; ^9^Heidelberg Institute of Global Health (HIGH), Heidelberg University Hospital, Heidelberg, Germany; ^10^Department of Infectious Diseases & Tropical Medicine, Ludwig Maximilians University of Munich, Munich, Germany; ^11^Faculty of Medicine, Max Von Pettenkofer Institute, Ludwig Maximilians University of Munich, Munich, Germany; ^12^German Center for Infection Research (DZIF), African Partner Institution, Lambaréné, Gabon; ^13^Kumasi Centre for Collaborative Research in Tropical Medicine (KCCR), Kumasi, Ghana

**Keywords:** antimicrobial resistance, sub-Saharan Africa, fever, *Salmonella enterica*, methicillin-resistant *Staphylococcus aureus*, Enterobacterales, extended-spectrum beta-lactamase-(ESBL)

## Abstract

**Background:**

Antimicrobial resistance (AMR) thwarts the curative power of drugs and is a present-time global problem. We present data on antimicrobial susceptibility and resistance determinants of bacteria the WHO has highlighted as being key antimicrobial resistance concerns in Africa, to strengthen knowledge of AMR patterns in the region.

**Methods:**

Blood, stool, and urine specimens of febrile patients, aged between ≥ 30 days and ≤ 15 years and hospitalized in Burkina Faso, Gabon, Ghana, and Tanzania were cultured from November 2013 to March 2017 (Patients > 15 years were included in Tanzania). Antimicrobial susceptibility testing was performed for all Enterobacterales and *Staphylococcus aureus* isolates using disk diffusion method. Extended-spectrum beta-lactamase (ESBL) production was confirmed by double-disk diffusion test and the detection of *bla*_CTX–M_, *bla*_TEM_ and *bla*_SHV_. Multilocus sequence typing was conducted for ESBL-producing *Escherichia coli* and *Klebsiella pneumoniae*, ciprofloxacin-resistant *Salmonella enterica* and *S. aureus*. Ciprofloxacin-resistant *Salmonella enterica* were screened for plasmid-mediated resistance genes and mutations in *gyrA, gyrB, parC, and parE*. *S. aureus* isolates were tested for the presence of *mecA* and Panton-Valentine Leukocidin *(PVL)* and further genotyped by *spa* typing.

**Results:**

Among 4,052 specimens from 3,012 patients, 219 cultures were positive of which 88.1% (*n* = 193) were Enterobacterales and 7.3% (*n* = 16) *S. aureus*. The prevalence of ESBL-producing Enterobacterales (all CTX-M15 genotype) was 45.2% (14/31; 95% CI: 27.3, 64.0) in Burkina Faso, 25.8% (8/31; 95% CI: 11.9, 44.6) in Gabon, 15.1% (18/119; 95% CI: 9.2, 22.8) in Ghana and 0.0% (0/12; 95% CI: 0.0, 26.5) in Tanzania. ESBL positive non-typhoid *Salmonella* (*n* = 3) were detected in Burkina Faso only and methicillin-resistant *S. aureus* (*n* = 2) were detected in Ghana only. While sequence type (ST)131 predominated among ESBL *E. coli* (39.1%;9/23), STs among ESBL *K. pneumoniae* were highly heterogenous. Ciprofloxacin resistant nt *Salmonella* were commonest in Burkina Faso (50.0%; 6/12) and all harbored *qnrB* genes. *PVL* were found in 81.3% *S. aureus*.

**Conclusion:**

Our findings reveal a distinct susceptibility pattern across the various study regions in Africa, with notably high rates of ESBL-producing Enterobacterales and ciprofloxacin-resistant nt *Salmonella* in Burkina Faso. This highlights the need for local AMR surveillance and reporting of resistances to support appropriate action.

## Introduction

Fever, caused by bacterial, viral, fungal, and parasitic pathogens, is a leading complaint presented at healthcare centers in sub-Saharan Africa (sSA) (Feikin et al., 2011; Hogan et al., 2018). Ideally, effective individual treatment involves identifying the infectious agents through cultures, serological and molecular tests and subsequently targeting them with appropriate antimicrobials (WHO, 2013). However, in several cases empirical treatment needs to be initiated before microbiological diagnostics to avert potential complications and to improve patient outcome (Aber et al., 2007). The inadequate diagnostic infrastructure in most African countries (Petti et al., 2006; Elbireer et al., 2011; Kapasi et al., 2016) further complicates targeted antimicrobial therapy resulting in heavy reliance on empiric judgement at hospitals, particularly those in remote areas.

As antimicrobial resistance (AMR) increases across the globe (Klein et al., 2018), adapting guidelines for empiric therapy to the changing drug susceptibility pattern is needed to allow better antimicrobial prescription decisions. At present, infections with AMR pathogens account for approximately 700,000 global deaths per year, and are estimated to cause up to 10 million annual deaths by 2050, with Africa expected to be one of the hardest hit continents (O’Neill, 2014). The mounting threat notwithstanding, only a few studies have so far addressed the growth of AMR in middle and low-income countries (Williams et al., 2018). Not surprisingly, investigations have indicated that guidelines on empirical antibiotic therapy in these countries are seldom if ever, formulated in view of regional microbiology and drug susceptibility patterns. For example, Elias et al. showed that only a third of recommendations on empirical antibiotic use for community-acquired pneumonia, urinary tract infections, acute otitis media, rhinosinusitis and pharyngitis considered the extent of local resistance, mostly in high-income settings (Elias et al., 2017). Enhancing understanding of the actual epidemiology of the local resistance landscape, on a routine basis, is therefore critical for adequate treatment guidelines, antimicrobial control policies and appropriate action plans in the regions. Although effort has been made by the WHO to foster national AMR surveillance systems in low-and middle-income countries by introducing GLASS (Global Antimicrobial Resistance Surveillance System) (WHO, 2015; Seale et al., 2017) only a few countries in sSA have functional AMR surveillance systems and for those that do, data from outside the major cities are scarce (GLASS | Global antimicrobial resistance surveillance system (GLASS) report, WHO, 2017).

This multicentre surveillance in rural and semi-urban Burkina Faso, Gabon, Ghana and Tanzania aimed to identify the causative pathogen for bloodstream-, urinary tract-, and gastrointestinal tract infections among hospitalized febrile patients with a focus on carbapenem-resistant and Extended-spectrum beta-lactamase (ESBL)-producing Enterobacterales, fluoroquinolone-resistant *Salmonella enterica* and methicillin-resistant *Staphylococcus aureus*, declared as WHO priority AMR pathogens. Results will be used to guide physicians and public health experts on local empiric antibiotic treatment decisions and hospital medication guidelines.

## Materials and Methods

### Study Population and Study Sites

The study was conducted among patients with an axillary or rectal or tympanic temperature of ≥ 38°C, admitted at hospitals in Burkina Faso, Gabon, Ghana and ambulatory patients in Tanzania from November 2013 to March 2017. Participants were between ≥ 30 days and ≤ 15 years of age from all sites, except for Tanzania where patients above 15 were also included. The largest among the hospitals (250 beds), is Agogo Presbyterian Hospital (APH) situated in the capital of Asante-Akim North District in Ghana. Its catchment area has an estimated 149,491 inhabitants^1^. Albert Schweitzer Hospital (ASH) is in Lambaréné, Gabon. As of 2017, the facility had 150 beds and served around 50,000 people a year (Dr. Schweitzer’s Hospital Fund, 2016). Nouna District Hospital (NDH) is located in the Kossi province, North West of Burkina Faso, with a total population of 374,239 inhabitants and 50 primary health care facilities^2^. The NDH has a bed capacity of 140, of which 50 are in the pediatric unit. In the Mbeya region, two health institutions were used: the Matema Lutheran Hospital, which is situated in a rural area in the Kyela district at the shore of Lake Nyassa and the Kiwanja Mpaka clinic which is in urban Mbeya, a town of 500,000 inhabitants.

### Detection and Identification of Pathogens

Specimens were collected from all patients at admission following physical examination by a medical practitioner and preceding drug administration. Three milliliters (ml) of blood was drawn from each child and 8–10 milliliters from adults. Each blood sample was inoculated into an aerobic paediatric/adult blood culture bottle (BACTEC (Peds) Plus Culture Vials, (Becton Dickinson, Germany) and processed using a BACTEC 9050 blood culture system (Becton Dickinson, Germany) according to the manufacturer’s instructions. Positive blood cultures were sub-cultivated on Columbia blood agar, chocolate agar, and MacConkey agar (all Oxoid, Basingstoke, United Kingdom) for species identification and antimicrobial susceptibility testing. If urinary tract infection signs (painful urination, renal angle tenderness, elevated leucocytes or nitrite in urine dipstick) were manifest, 2 ml urine was obtained from patients and plated on Chromogenic UTI Medium (Oxoid, Basingstoke, United Kingdom). From cases presenting with diarrhea, defined as three or more loose stools in 24 h, a tea-spoon size of stool was screened for *S. enterica* and *Shigella* spp. Samples were enriched overnight on selenite F broth and then cultured on xylose lysine deoxycholate agar (Becton Dickinson, Germany) and MacConkey agar.

Incubations were done overnight at 35–37°C. All bacterial colonies were further identified by standard biochemical methods using the Analytical profile Index test (API 20E, bioMérieux, Marcy L’Etoile, France). Environmental bacteria and bacteria belonging to the skin flora (e.g., coagulase-negative Staphylococci except *S. saprophyticus* in urine and *S. lugdunensis* in blood culture, *Corynebacterium* species, and *Bacillus* species) were considered as contaminants. All isolates were sent to Germany on dry ice for species confirmation by MALDIToF MS (Bruker Daltonics, Bremen, Germany) and subtyping analysis.

### Antimicrobial Susceptibility Testing

For all Enterobacterales and *S. aureus*, antimicrobial susceptibility testing was performed using Kirby-Bauer disk diffusion method on Mueller-Hinton agar according to the 2019 European Committee on Antimicrobial Susceptibility Testing (EUCAST) guidelines (EUCAST: EUCAST, 2019). Enterobacterales were tested against ampicillin, cefotaxime, ceftazidime, meropenem, ciprofloxacin, gentamicin, tigecycline and sulfamethoxazole/trimethoprim (all Oxoid, Basingstoke, United Kingdom). Ciprofloxacin resistance was defined as a MIC > 0.06 mg/L for *S. enterica*, confirmed by E-test (Oxoid, Basingstoke, United Kingdom) and a MIC > 1 mg/L for other Enterobacterales. A positive ESBL phenotype was confirmed by the double-disk diffusion test with cefotaxime and ceftazidime alone and in combination with clavulanic acid (Becton, Dickinson and Company, Sparks, MD, United States) as described before by the EUCAST (EUCAST, 2020: Resistance mechanisms). *S. aureus* isolates were tested against penicillin, cefoxitin, clindamycin, erythromycin, ciprofloxacin, tetracycline, sulfamethoxazole/trimethoprim, tigecycline, gentamicin, rifampicin, linezolid, teicoplanin and vancomycin (all Oxoid, Basingstoke, United Kingdom).

### ESBL Genotyping and Ciprofloxacin Resistance Mechanisms

All isolates with ESBL phenotypes were screened for the presence of *bla*_*rm CTXM*_, *bla*_*rm TEM*_ and *bla*_*rm SHV*_ genes by polymerase chain reaction (PCR) and sequenced as previously described. (Belmar Campos et al., 2014). Group specific primers were used to distinguish *bla*_*rm CTXM*_ positive isolates (Belmar Campos et al., 2014). The resulting sequences were compared with known sequences using NCBI BLAST^3^ and the Lahey Clinic Database^4^. Screening of ciprofloxacin non-susceptible *S. enterica* isolates for point mutations in DNA gyrase and/or DNA topoisomerase IV genes *gyrA*, *gyrB*, *parC*, *pare*, and plasmid-mediated *aac(6’)Ib(-cr)*, *qnrA*, *qnrB*, *qnrC*, *qnrD*, and *qnrS* resistance genes was performed as previously described (Chau et al., 2007; Pfeifer et al., 2009; Pietsch et al., 2017).

### *Spa*-Typing and Multilocus Sequence Typing (MLST)

MLST was conducted for all *S. aureus*, ESBL-producing *Escherichia coli* and *Klebsiella pneumoniae* and ciprofloxacin-resistant *S. enterica* according to previously published loci protocols (Diancourt et al., 2005; Wirth et al., 2006). *S. aureus* isolates were genotyped by *spa*-typing as described before (Eibach et al., 2017) with *spa*-types being determined using the StaphType software and the Ridom SpaServer^5^.

### Epidemiological Analysis

Categorical variables were displayed with percentages, and continuous data with the median and interquartile range (IQR). Data analysis was performed with Stata version 14 software (StataCorp, College Station, Texas). Ninety five percent confidence intervals were calculated using the exact Clopper-Pearson method.

### Ethical Considerations

The study protocol was approved by the Institutional Review board of Nouna Research Centre and the National Ethics Committee for Health Research in Burkina Faso, the Comité d’Ethique Institutionel in Lambaréné, Gabon, the Committee on Human Research, Publications and Ethics from the School of Medical Science, Kwame Nkrumah University of Science and Technology, Kumasi, Ghana, the Ethics committee of the medical faculty of the Ludwig Maximilians University in Munich, the Tanzanian National Research and Ethics committee, and the Mbeya Medical Research and Ethics Committee for Tanzania and the Ethics Committee of the Ärztekammer Hamburg, Germany. All participants were informed about the study’s purpose and procedures. Written informed consents were obtained from all participants above 18 years. In older children, written informed assent and consent was obtained from the patients and their parents, respectively. In case of infants, the parents or legal guardian provided written informed consent prior to enrolment.

## Results

Altogether, the study recorded 3,012 fever cases aged between 30 days and 81 years from Burkina Faso, Gabon, Ghana and Tanzania. Patients above 15 years were only included in Tanzania (*n* = 188). For all pediatric study sites, the median patient age was 2.0 years (IQR 0.9–4.0) and that of Tanzania was 9.1 (IQR 3.8–24.5). The sex ratio was balanced (Female: 48.2%, *n* = 1,452) and the median temperature was 38.9°C (IQR 38.4–39.5°C). Possible bacterial causes of fever were analyzed in 3423 blood, 629 urine and 412 stool samples obtained. Table 1 provides demographic information on the individuals recruited.

**TABLE 1:** Participants overview.

Country	Burkina Faso	Gabon	Ghana	Tanzania	Total
Patients, *N*	478	600	1238	696	3012
Age (median; IQR)	1.0; 0.3–2.0	2.4; 1.0–5.7	2.0; 1.0–4.0	6.7; 3.4–17.6	2.4; 1.0–5.6
Female, *N* (%)	221 (46.2)	280 (46.7)	561 (45.3)	390 (56.0)	1452 (48.2)
Temperature (median; IQR)	38.8; 38.4–39.3	39.0; 38.4–39.7	39.0; 38.5–39.6	38.9; 38.4–39.4	38.9; 38.4–39.5

*IQR, interquartile range; N, sample size.*

### Bacterial Cultures

Overall, 219 cultures were positive. The highest culture positivity rate was found in urine (15.4%; 97/629) (95% CI: 12.7, 18.5), followed by blood (3.3%; 113/3009) (95% CI: 3.1, 4.5) and stool samples (2.2%; 9/412) (95% CI: 1.0, 4.1). The positive urine cultures were most frequently detected in Ghana (26.5%; 63/238) (95% CI: 21.0, 32.6), then Burkina Faso (9.5%; 10/105) (95% CI: 4.7, 16.8) and Gabon (8.4%; 24/286) (95% CI: 5.5, 12.2). Positive blood cultures were most prevalent among samples from Burkina Faso (5.6%; 27/482) (95% CI: 3.7, 8.0), then Ghana (4.9%; 61/1238) (95% CI: 3.8, 6.3) and Gabon (1.4%; 8/593) (95% CI: 0.6, 2.6). For Tanzania, blood culture positivity was 3.7% (7/188) among participants above 15 years and 2.0% (10/508) among children up to 15 years of age. Stool cultures for *Salmonella/Shigella* infections were commonest in Burkina Faso (5.6%; 1/18) (95% CI: 0.1, 27.3), followed by Gabon (3.3%; 3/91) (95% CI: 0.7, 9.3) and Ghana (2.2%; 5/232) (95% CI: 0.7, 5.0). No pathogenic bacterium was isolated in stool samples from Tanzania. Uropathogens detected were mainly *E. coli* (62.9%; *n* = 61) and *K. pneumoniae* (19.6%; *n* = 19). Bloodstream bacteria included *S. enterica* (72.9%; *n* = 70), *E. coli* (9.4%; *n* = 9), *S. aureus* (8.3%; *n* = 8) and *K. pneumoniae* (3.1%; *n* = 3). Gastrointestinal pathogens were *S. enterica* (88.9%; *n* = 8) and *Shigella sonnei* (11.1%; *n* = 1). All cultures processed and country distribution of the species isolated are presented in Table 2.

**TABLE 2:** Cultures processed and country distribution of the bacteria isolates.

	Burkina Faso	Gabon	Ghana	Tanzania	All countries
Number of all samples processed, *n*	605	970	1708	769	4052
All bacteria isolated, *n* (%)	38 (6)	35 (4)	129 (8)	17 (2)	219 (5)
Blood samples processed, *n*	482	593	1238	696	3009
Bacteria isolated from blood, *n* (%)	27 (6)	8 (1)	61 (5)	17 (2)	113 (4)
*Enterococcus faecalis*	4 (15)	0 (0)	0 (0)	0 (0)	4 (4)
*Escherichia coli*	7 (26)	1 (13)	1 (2)	2 (12)	11 (10)
*Klebsiella oxytoca*	0 (0)	0 (0)	1 (2)	0 (0)	1 (1)
*Klebsiella pneumoniae*	1 (4)	1 (13)	1 (2)	0 (0)	3 (3)
Nt *Salmonella*	10 (37)	4 (50)	55 (90)	0 (0)	69 (61)
*Salmonella* Typhi	1 (4)	0 (0)	0 (0)	10 (59)	11 (10)
*Serratia marcescens*	1 (4)	0 (0)	0 (0)	0 (0)	1 (1)
*Staphylococcus aureus*	3 (11)	2 (25)	3 (5)	5 (29)	13 (12)
Stool samples processed, *n*	18	91	232	71	412
Bacteria isolated from stool *n* (%)	1 (6)	3 (3)	5 (2)	0 (0)	9 (2)
Nt *Salmonella*	1 (100)	2 (67)	5 (100)	0 (0)	8 (89)
*Shigella sonnei*	0 (0)	1 (33)	0 (0)	0 (0)	1 (11)
Urine samples processed, *n*	105	286	238	0	629
Bacteria isolated from urine *n* (%)	10 (10)	24 (8)	63 (26)	0 (0)	97 (15)
*Acinetobacter baumannii* complex	0 (0)	1 (4)	1 (2)	0 (0)	2 (2)
*Enterobacter cloacae*	0 (0)	0 (0)	1 (2)	0 (0)	1 (1)
*Enterococcus faecium*	0 (0)	0 (0)	3 (5)	0 (0)	3 (3)
*Escherichia coli*	8 (80)	10 (42)	43 (68)	0 (0)	61 (63)
*Klebsiella pneumoniae*	1 (10)	7 (29)	11 (17)	0 (0)	19 (20)
*Proteus mirabilis*	0 (0)	2 (8)	1 (2)	0 (0)	3 (3)
*Proteus penneri*	0 (0)	1 (4)	0 (0)	0 (0)	1 (1)
*Proteus vulgaris*	0 (0)	1 (4)	0 (0)	0 (0)	1 (1)
*Pseudomonas aeruginosa*	0 (0)	0 (0)	1 (2)	0 (0)	1 (1)
Nt *Salmonella*	1 (10)	1 (4)	0 (0)	0 (0)	2 (2)
*Staphylococcus aureus*	0 (0)	1 (4)	2 (3)	0 (0)	3 (3)

*n, sample size; nt Salmonella, non-typhoid Salmonella.*

### ESBL in Enterobacterales Across Countries

Enterobacterales accounted for 88.1% (193/219) of the positive cultures, with *S. enterica* (41.1%; *n* = 90), *E. coli* (32.9%; *n* = 72) and *K. pneumoniae* (10.0%; *n* = 22) being the most prevalent. The most active antibiotics against these bacteria were meropenem (1.6%; *n* = 3 intermediate resistance), tigecycline (7.8%; *n* = 15 resistance) and ciprofloxacin (19.2%; *n* = 37 resistance). Resistance was highest against ampicillin (67%; *n* = 130), trimethoprim-sulfamethoxazole (62%; *n* = 119) and gentamicin (50%; *n* = 96). Table 3 shows resistance patterns of all the Enterobacterales to the antibiotics tested. ESBL-producing organisms constituted 20.7% (*n* = 40) of all Enterobacterales with the highest rate being among *K. pneumoniae* (63.6%; 14/22) and *E. coli* (31.9%; 23/72), and lowest among *S. enterica* (3.8%; 3/79). In decreasing order, their proportions were: 45.2% (14/31) (95% CI: 27.3, 64.0) in Burkina Faso, 25.8% (8/31) (95% CI: 11.9, 44.6) in Gabon and 15.1% (18/119),(95% CI: 9.2, 22.8) in Ghana. None was found in Tanzania (Table 4). The ESBL-producing Enterobacterales across all countries harbored only *bla*CTX-M15 ESBL genes. Thirteen MLST sequence types (ST) were identified among ESBL *E. coli* and 13 among ESBL *K. pneumoniae.* The set of ST found was unique for each country (Table 5), except for ST 131, which was found among nine ESBL *E. coli*, mainly in Burkina Faso (*n* = 5), Gabon (*n* = 1) and Ghana (*n* = 3).

**TABLE 3:** Drug resistance patterns of the Enterobacterales.

	Ampicillin	Cefotaxime	Ceftazidime	Ciprofloxacin	Gentamicin	Meropenem	SXT	Tigecycline
	
	*n* (*n*/*N*%)	*n* (*n*/*N*%)	*n* (*n*/*N*%)	*n* (*n*/*N*%)	*n* (*n*/*N*%)	*n* (*n*/*N*%)	*n* (*n*/*N*%)	*n* (*n*/*N*%)
All Enterobacterales (*N* = 193)	130 (67)	40 (21)	40 (21)	37 (19)	96 (50)	3 (2)	119 (62)	15 (8)
*Nt Salmonella* (*N* = 79)	32 (41)	3 (4)	3 (4)	8 (10)	63 (80)	0 (0)	34 (43)	2 (3)
*Escherichia coli* (*N* = 72)	59 (82)	23 (32)	23 (32)	21 (29)	19 (26)	0 (0)	58 (81)	2 (3)
*Klebsiella pneumoniae* (*N* = 22)	22 (100)	14 (64)	14 (64)	6 (27)	13 (59)	0 (0)	13 (59)	6 (27)
*Salmonella* Typhi (*N* = 11)	10 (91)	0 (0)	0 (0)	2 (18)	0 (0)	3 (27)	10 (91)	0 (0)
*Proteus mirabilis* (*N* = 3)	2 (67)	0 (0)	0 (0)	0 (0)	0 (0)	0 (0)	2 (67)	3 (100)
*Enterobacter cloacae* (*N* = 1)	1 (100)	0 (0)	0 (0)	0 (0)	0 (0)	0 (0)	0 (0)	0 (0)
*Klebsiella oxytoca* (*N* = 1)	1 (100)	0 (0)	0 (0)	0 (0)	0 (0)	0 (0)	0 (0)	0 (0)
*Proteus penneri* (*N* = 1)	1 (100)	0 (0)	0 (0)	0(0)	0 (0)	0 (0)	0 (0)	1 (100)
*Proteus vulgaris* (*N* = 1)	1 (100)	0 (0)	0 (0)	0 (0)	0 (0)	0 (0)	0 (0)	1 (100)
*Serratia marcescens* (*N* = 1)	1 (100)	0 (0)	0 (0)	0 (0)	0 (0)	0 (0)	1 (100)	0 (0)
*Shigella sonnei* (*N* = 1)	0 (0)	0 (0)	0 (0)	0 (0)	1 (100)	0 (0)	1(100)	0 (0)
ESBL-producing Enterobacterales (*N* = 40)	40 (100)	40 (100)	40 (100)	27 (68)	31 (78)	0 (0)	36 (90)	3 (8)
*Escherichia coli* (*N* = 23)	23 (100)	23 (100)	23 (100)	18 (78)	15 (65)	0 (0)	22 (96)	0 (0)
*Klebsiella pneumoniae* (*N* = 14)	14 (100)	14 (100)	14 (100)	6 (43)	13 (93)	0 (0)	11 (79)	3 (21)
Nt *Salmonella* (*N* = 3)	3 (100)	3 (100)	3 (100)	3 (100)	3 (100)	0 (0)	3 (100)	0 (0)

*N,n, sample size; Nt Salmonella, non-typhoid Salmonella; SXT, trimethoprim-sulfamethoxazole; ESBL, extended-spectrum beta-lactamase.*

**TABLE 4:** Prevalence of resistances among the isolated bacteria to commonly used antibiotics.

		Burkina Faso n (%)	Gabon n (%)	Ghana n (%)	Tanzania n (%)	All countries n (%)
**Enterobacterales**		***N* = 31**	***N* = 31**	***N* = 119**	***N* = 12**	***N* = 193**
	Ampicillin	28 (90)	19 (61)	72 (61)	11 (92)	130 (67)
	Cefotaxime	14 (45)	8 (26)	18 (15)	0 (0)	40 (21)
	Ceftazidime	14 (45)	8 (26)	18 (15)	0 (0)	40 (21)
	Meropenem	0 (0)	0 (0)	0 (0)	3 (25)	3 (2)
	Gentamicin	13 (42)	8 (26)	75 (63)	0 (0)	96 (50)
	Ciprofloxacin	16 (52)	4 (13)	16 (13)	1 (8)	37 (19)
	Tigecycline	0 (0)	7 (23)	8 (7)	0 (0)	15 (8)
	SXT	31 (100)	13 (42)	66 (55)	9 (75)	119 (62)
	Confirmed ESBL	14 (45)	8 (26)	18 (15)	0 (0)	40 (21)
***Staphylococcus aureus***		***N* = 3**	***N* = 3**	***N* = 5**	***N* = 5**	***N* = 16**
	Gentamicin	0 (0)	0 (0)	0 (0)	0 (0)	0 (0)
	Ciprofloxacin	0 (0)	0 (0)	0 (0)	0 (0)	0 (0)
	Tigecycline	0 (0)	0 (0)	0 (0)	0 (0)	0 (0)
	STX	2 (67)	3 (100)	2 (40)	5 (100)	12 (75)
	Penicillin	3 (100)	3 (100)	5 (100)	5 (100)	16 (100)
	Cefoxitin	0 (0)	0 (0)	2 (40)	0 (0)	2 (13)
	Erythromycin	1 (33)	0 (0)	1 (20)	0 (0)	2 (13)
	Clindamycin	0 (0)	0 (0)	1 (20)	0 (0)	1 (6)
	Teicoplanin	0 (0)	0 (0)	0 (0)	0 (0)	0 (0)
	Vancomycin	0 (0)	0 (0)	0 (0)	0 (0)	0 (0)
	Linezolid	0 (0)	0 (0)	0 (0)	0 (0)	0 (0)
	Rifampicin	0 (0)	0 (0)	0 (0)	0 (0)	0 (0)
	Tetracyclin	3 (100)	1 (33)	4 (80)	0 (0)	8 (50)
***Pseudomonas aeruginosa***		***N* = 0**	***N* = 0**	***N* = 1**	***N* = 0**	***N* = 1**
	Meropenem	0 (0)	0 (0)	0 (0)	0 (0)	0 (0)

*N,n, sample size; SXT, trimethoprim-sulfamethoxazole; ESBL, extended-spectrum beta-lactamase.*

**TABLE 5:** Multilocus sequence types for ESBL-producing *Escherichia coli* and *Klebsiella pneumoniae* isolates by country.

	Burkina Faso	Gabon	Ghana	Tanzania	All countries
**ESBL *E. coli* sequence types (ST)**	***N* = 9**	***N* = 2**	***N* = 12**	***N* = 0**	***N* = 23**
ST131	5	1	3	NA	9
ST410	0	0	3	NA	3
ST10	0	0	2	NA	2
ST167	1	0	0	NA	1
ST3052	0	1	0	NA	1
ST38	0	0	1	NA	1
ST410	1	0	0	NA	1
ST617	1	0	0	NA	1
ST617	0	0	1	NA	1
ST648	0	0	1	NA	1
ST93	0	0	1	NA	1
Non-assigned ST	1	0	0	NA	1
**ESBL *K. pneumoniae* sequence types (ST)**	***N* = 2**	***N* = 6**	***N* = 6**	***N* = 0**	***N* = 14**
ST14	2	0	0	NA	2
ST1031	0	1	0	NA	1
ST1072	0	1	0	NA	1
ST1430	0	1	0	NA	1
ST147	0	1	0	NA	1
ST1891	0	1	0	NA	1
ST215	0	0	1	NA	1
ST2734	0	0	1	NA	1
ST307	0	0	1	NA	1
ST3248	0	1	0	NA	1
ST36	0	0	1	NA	1
ST39	0	0	1	NA	1
ST530	0	0	1	NA	1

*ST, sequence type; N, sample size.*

### Resistance to Ciprofloxacin Among Enterobacterales

The proportion of Enterobacterales with reduced susceptibility to ciprofloxacin was notably higher in Burkina Faso (51.6%; 16/31) (95% CI: 33.1, 69.8), relative to 12.9% (4/31) (95% CI: 3.6, 29.8) in Gabon, 13.4% (16/119) (95% CI: 7.9, 20.9) in Ghana. In Tanzania, 8.3% Enterobacterales were resistant to ciprofloxacin (Table 4). Among nt *Salmonella*, ciprofloxacin resistance was 50.0% (6/12, (95% CI: 21.1, 78.9) in Burkina Faso, 3.3% (2/60) (95% CI: 0.4, 11.5) in Ghana and 0.0% (95% CI: 0.0, 41.0) in Gabon (0/7). No ciprofloxacin resistant nt Salmonella was found in Tanzania. All isolates from Burkina Faso were ST 313 and harbored *qnrB* genes (5 blood, 1 stool) while both isolates (blood) from Ghana had a single *gyrA* mutation at position D87G. A ciprofloxacin-resistant *Salmonella* Typhi was isolated in a blood sample from Burkina Faso. The pathogen had a *gyrA* double mutations (D87G and E113G). A second bloodstream ciprofloxacin-resistant *S.* Typhi with a *gyrA* mutation at position E113G and a *gyrB* mutation S464Y was found in Tanzania. Among nt *Salmonella*, ESBL production and ciprofloxacin resistance concurrence was observed in a single stool and two blood isolates from Burkina Faso only. No *parC*, *parE* mutations as well as *aac (6’) Ib(-cr)*, *qnrA*, *qnrC*, *qnrD* and *qnrS* genes were found (Table 6).

**TABLE 6:** Distribution of mutations and genes conferring ciprofloxacin resistance among *Salmonella enterica.*

ID	country	*Salmonella enterica*	Sequence type	MIC (g/L)	Mutation in *gyrA*	Mutation in *gyrB*	plasmid mediated genes	ESBL
118	Tanzania	*S*. Typhi	ST1	0.19	E133G	S464Y	Negative	Negative
700613	Ghana	nt *Salmonella*	ST11	0.064	D87G	no	Negative	Negative
701845	Ghana	nt *Salmonella*	ST11	0.064	D87G	no	Negative	Negative
A00-Iso02141	Burkina Faso	nt *Salmonella*	ST313	0.38	no	no	*qnrB*	ESBL
A06-Iso02144	Burkina Faso	nt *Salmonella*	ST313	1	no	no	*qnrB*	Negative
A56-Iso02149	Burkina Faso	nt *Salmonella*	ST313	0.75	no	no	*qnrB*	Negative
C00-Iso02162	Burkina Faso	nt *Salmonella*	ST313	0.25	no	no	*qnrB*	ESBL
C04-Iso02164	Burkina Faso	nt *Salmonella*	ST313	0.25	no	no	*qnrB*	ESBL
D00-Iso02167	Burkina Faso	*S*. Typhi	ST2	0.064	D87G, E133G	no	Negative	Negative
D01-Iso02170	Burkina Faso	nt *Salmonella*	ST313	1	no	no	*qnr B*	Negative

*ID, patient identification number; S. Typhi, Salmonella Typhi; nt Salmonella, non-typhoid Salmonella; ST, sequence type; MIC, minimum inhibitory concentration; ESBL, extended-spectrum beta-lactamase.*

### Methicillin-Resistant *S. aureus*

A total of 16 *S. aureus*, constituting 7.3% of all positive cultures, were isolated from thirteen blood and three urine cultures, mostly from Tanzania (*n* = 5) and Ghana (*n* = 5) (Table 2). The isolates belonged to six *spa*-types and five STs. ST 152 was the most common clone, which accounted for 80.0% (4/5), 100% (3/3), 66.7% (2/3), and 20.0% (1/5) of *S. aureus* from Tanzania, Burkina Faso, Gabon and Ghana, respectively. The most frequent *spa*-types included t355 (50.0%; *n* = 8), followed by t186 (12.5%; *n* = 2) and t314 (12.5%; *n* = 2). Panton-Valentine Leukocidin (*PVL*) were found in 13 (81.3%) isolates, of which 10 (76.9%) were ST152. All *S. aureus* isolates from Tanzania and Burkina Faso, were *PVL* positive. Two MRSA were found in Ghana, both ST 88, *spa*-type t186, *mecA* positive and *PVL* negative (Table 7).

**TABLE 7:** Sequence types and *spa* types of *Staphylococcus aureus* isolates by country.

Country	Sequence Type (ST) (*n*)	spa types (*n*)	MRSA *n* (%)	*PVL n* (%)
Burkina Faso (*N* = 3)*	ST152 (3)	t314 (1), t355 (1), t4198 (1)	0(0)	3 (100)
Gabon (*N* = 3)*	ST152 (2), ST8 (1)	t355 (2), t1476 (1)	0(0)	2 (67)
Ghana (*N* = 5)*	ST121 (1), ST152 (1), ST15 (1), ST88 (2)	t314 (1), t4454 (1), t355 (1), t186 (2)	2(40)	3 (60)
Tanzania (*N* = 5)*	ST 152(4), ST88 (1)	t355 (4), t2526 (1)	0(0)	5 (100)

*No vancomycin resistance was observed.N,n, sample size; MRSA, methicillin-resistant Staphylococcus aureus; PVL, Panton-Valentine leucocidin.

## Discussion

The current study identifies Enterobacterales as a major cause of fever in the hospitals in rural sub-Saharan Africa and reports that resistance to widely used antibiotics including penicillins, cephalosporins and fluoroquinolones is common and geographically variable. We found unequal rates of ESBL-producing Enterobacterales, ranging from 45% (Burkina Faso) to 26% (Gabon) to 15%(Ghana). These proportions are in line with reports from similar studies in the respective study countries (Alabi et al., 2013; Eibach et al., 2016b; Ouedraogo et al., 2016). However, differing ESBL rates have been reported from previous studies conducted in large cities in the countries, suggesting that AMR varies within-country as well. (Obeng-Nkrumah et al., 2013; Yala et al., 2016; Kpoda et al., 2017). The regional differences may be due to ununiform methodological approaches adopted in the studies, such as, varying inclusion criteria (age groups, type of hospital, in-patient or out-patient, symptoms etc.). To allow better comparability of results and improve data validity, we underscore the significance of standardized AMR sampling and detection methods and harmonized interpretation procedures using tools such as the GLASS guidelines to this end (WHO, 2015).

Being the most frequently detected pathogen from blood cultures in the present study (70.8%; 69 nt *Salmonella*, 11 *S.* Typhi), resistance rates of *S. enterica* may support empirical treatment for invasive bloodstream infections in our study regions. Notably, ESBL nt *Salmonella* were found in blood and stool samples from Burkina Faso only, similar to what has been reported before (Maltha et al., 2014). No ESBL production was detected among *S.* Typhi. These low frequencies of ESBL-producing *S. enterica* indicate that the pathogens remain largely susceptible to the recommended third generation cephalosporins.

Remarkable regional differences were detected for ciprofloxacin-resistant nt *Salmonella*, with a frequency more than 10-folds higher observed in Burkina Faso compared to Ghana. A similar observation has previously been described (Eibach et al., 2016a). High levels of ciprofloxacin-resistant *S.* Typhi in Kenya and none in Central or West Africa have been reported. The authors postulated a spread of these resistances from the Indian subcontinent to East Africa (Al-Emran et al., 2016). We found ciprofloxacin-resistant *S.* Typhi in Tanzania (1/10) and Burkina Faso (1/1), hinting at a possible dissemination to West Africa that needs to be monitored closely.

We found high ESBL rates among *E. coli* and *K. pneumoniae.* These organisms being important sources of transferrable antibiotic resistance and nosocomial outbreaks, the observed rates raise concern in countries with limited antibiotic treatment options (Eibach et al., 2016b). For *K. pneumoniae* a clear geographical segregation with no overlapping STs or any predominant *K. pneumoniae* strains across the participating countries was observed. We found ST 131 *E. coli* among blood samples in Burkina Faso and urine samples in Ghana, Burkina Faso and Gabon. ESBL ST 131 *E. coli* has been described as a major cause of extraintestinal infections worldwide (e.g., UTI, bloodstream infections, meningitis, soft tissue infections) (Nicolas-Chanoine et al., 2014).

This study also reports methicillin resistance in two *S. aureus* isolates from Ghana. Equally low numbers of *S. aureus* were found in Burkina Faso, Gabon and Tanzania although none was MRSA.

The geographical disparity of AMR levels observed in our study may be associated with socio-economic and health factors, as described in other studies. In one such study, a systematic segregation of high-income countries in Europe, North America, Oceania from low-income countries in Africa, Asia, South America according to AMR gene abundance was demonstrated (Hendriksen et al., 2019). The present study reveals a separation of countries within sSA with Burkina Faso, which ranks very low (rank 182) in the human development report showing higher resistance levels than Gabon and Ghana, ranking at 115 and 142, respectively (UNDP, 2019). It is notable that these findings hardly provide a representative basis but expose the need for expanding region-specific surveillance networks for AMR in African countries. On this matter, a recent progress report shows that 11/31 countries in sSA, Ghana included, have a national action plan (NAP) approved by government, with an operational plan and monitoring arrangements. Tanzania is already in the implementation phase while NAPs in Burkina Faso and Gabon have just recently been developed (WHO, 2019).

Some limitations of this study need to be addressed. First, there was no age restriction for participants from Tanzania whereas the other study sites included only children, thus, comparability of results from Tanzania to those from other countries was undermined. In some instances, the culture positivity rate was low. Easy availability of non-prescription antibiotics (Auta et al., 2019) and self-medicating prior to hospitalization (Okeke et al., 2007; Kariuki and Dougan, 2014) may have caused the bacterial cultures to remain negative. Also, drawing sufficient quantities of blood samples from children was difficult, hence the low positivity rates. In this light, the proportions reported here should be interpreted in view of the isolate numbers. Also important is that our study was performed in single hospitals and this does not necessarily reflect the antimicrobial resistance trends in other regions within the countries.

## Conclusion

This study reveals high occurrence of ESBLs and ciprofloxacin-resistant Enterobacterales in clinical samples from regions in sSA. Further effort to enhance reporting of regional epidemiological AMR data is needed for evidence-based local treatment guidelines. On a national level, initiatives to scale up antimicrobial surveillance systems ought to be supported in the African countries to drive interventions aimed at limiting the dissemination of drug resistant pathogens. On an encouraging note, the study found no resistance to carbapenems, meaning that these antibiotics, although costly and hardly available, hold potential as an alternative therapy for ESBL-producing Enterobacterales. Nevertheless, vigilance to detect emerging carbapenemase-producing pathogens, in time, is required.

## Data Availability Statement

The raw data supporting the conclusions of this article will be made available by the authors, without undue reservation, to any qualified researcher.

## Ethics Statement

The studies involving human participants were reviewed and approved by the Institutional Review board of Nouna Research Centre and the National Ethics Committee for Health Research in Burkina Faso, the Comité d’Ethique Institutionel in Lambaréné, Gabon, the Committee on Human Research, Publications and Ethics from the School of Medical Science, Kwame Nkrumah University of Science and Technology, Kumasi, Ghana, the Ethics committee of the medical faculty of the Ludwig Maximilians University in Munich, the Tanzanian National Research and Ethics committee, and the Mbeya Medical Research and Ethics Committee for Tanzania and the Ethics Committee of the Ärztekammer Hamburg, Germany. All participants were informed about the study’s purpose and procedures. Written informed consents were obtained from all participants above 18 years. In older children, written informed assent and consent was obtained from the patients and their parents, respectively. In case of infants, the parents or legal guardian provided written informed consent prior to enrolment.

## Author Contributions

DE, JM, AA, BM, ASo, NH, and ML designed and coordinated the study. NN, JF, JH, CM, LS, BC, EO-D, and ASi conducted and supervised the fieldwork. FS, AW, DD, and DE supervised the laboratory work. EL and RM performed the epidemiological and statistical analysis. RM, EL, and DE wrote the first draft of the manuscript. All authors read and approved the final manuscript.

## Conflict of Interest

The authors declare that the research was conducted in the absence of any commercial or financial relationships that could be construed as a potential conflict of interest.

## Footnotes

1 https://www.statsghana.gov.gh/

2 http://www.insd.bf/n/

3 https://blast.ncbi.nlm.nih.gov/Blast.cgi

4 https://omictools.com/the-lahey-clinic-database-tool

5 http://spaserver.ridom.de/
